# Extraocular muscle Diffusion Weighted Imaging as a quantitative metric of posterior orbital involvement in thyroid associated orbitopathy

**DOI:** 10.1186/s13244-024-01757-x

**Published:** 2024-08-01

**Authors:** Nicole M. George, Claire Feeney, Vickie Lee, Parizad Avari, Amina Ali, Gitta Madani, Ravi Kumar Lingam, Kunwar S. Bhatia

**Affiliations:** 1 Imperial College London, School of Medicine, London, UK; 2 Royal Surrey County Hospital, Guildford, UK; 3 Department of Endocrinology, Imperial College Healthcare NHS Trust, London, UK; 4 Western Eye Hospital, Imperial College Healthcare NHS Trust, London, UK; 5 Department of Radiology, Charing Cross Hospital, Imperial College Healthcare NHS Trust, London, UK; 6 Department of Radiology, Northwick Park & Central Middlesex Hospital, London Northwest University Healthcare NHS Trust, London, UK

**Keywords:** Diffusion magnetic resonance imaging, Echo-planar imaging, Thyroid eye disease, Disease severity

## Abstract

**Objectives:**

The clinical activity score (CAS) and European severity scale (ESS) are established clinical tools to assess thyroid eye disease (TED) but are limited in terms of subjectivity and their reliability in non-Caucasian individuals, and can underestimate significant disease in the posterior orbit. Preliminary data from pilot studies have shown that diffusion-weighted imaging (DWI) using extraocular muscle (EOM) apparent diffusion coefficient (ADC) measurements may provide complementary information in TED. This study expands on previous research to assess for correlations between clinical scores and EOM-ADCs in stratifying disease activity and severity in a large patient cohort from an ethnically diverse population.

**Methods:**

A retrospective review of TED clinics between 2011 and 2021 identified 96 patients with a documented CAS and ESS and an orbital MRI that included DWI. From regions of interest manually placed on EOM bellies, the highest ADC was computed for each patient and analysed for correlations and associations with CAS and ESS using Spearman Rank correlation and Mann–Whitney *U* tests, and any potential discriminatory cut-offs using Receiver Operator Curve analyses. A *p*-value < 0.05 indicated statistical significance.

**Results:**

EOM-ADCs showed a positive association with CAS (*p* ≤ 0.001). EOM-ADCs were higher in sight-threatening compared to mild disease (*p* ≤ 0.01). A cut-off of 995 mm^2^/s achieved AUC = 0.7744, equating to 77% sensitivity and 67% specificity for discrimination between mild-moderate and sight-threatening disease.

**Conclusion:**

EOM-ADCs correlate with higher scores of disease severity and activity in TED. Besides providing quantitative data to support clinical tools, EOM-ADC cut-offs may identify patients at risk of developing sight-threatening diseases.

**Critical relevance statement:**

This study critically evaluates the limitations of conventional clinical assessment tools for TED and demonstrates the utility of DWI scans with ADC measurements in identifying active disease, offering valuable insights to advance clinical radiology practice.

**Key Points:**

- Conventional tools for TED assessment have subjective limitations.
- ADCs from non-echoplanar diffusion-weighted imaging correlate with clinical activity.
- Non-echoplanar diffusion-weighted imaging offers quantitative assessment to aid clinical practice reliability.

**Graphical Abstract:**

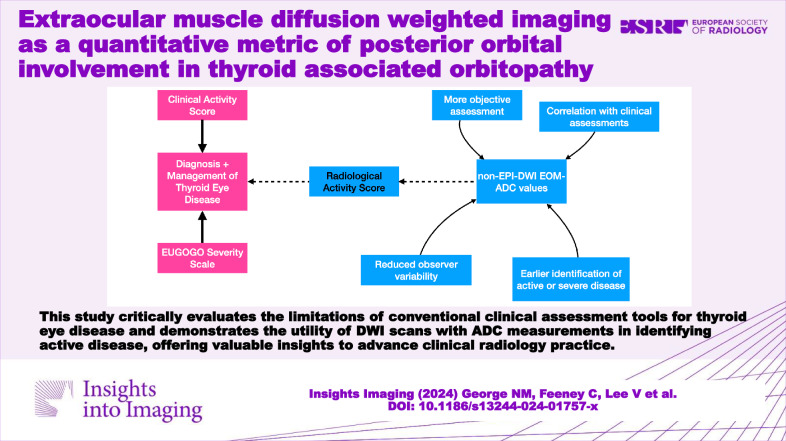

## Introduction

Thyroid eye disease (TED) is an autoimmune condition usually, but not invariably, associated with Graves’ hyperthyroidism. The underlying pathophysiology is complex but typically manifests with the expansion of orbital and periorbital tissues, especially intra and extraconal fat and extraocular muscles (EOMs) [1]. This disease follows a sequential time course lasting a few years in most patients, with a clinically active phase initially followed by an inactive chronic phase. Some degree of cosmetic disfigurement is almost universal in patients with moderate/severe disease, and sight-threatening disease can lead to diplopia and visual loss through corneal ulceration and optic nerve compression [1]. About one-third of patients benefit from timely immunosuppression during to active phase to modify the disease course and lessen residual disease burden [1]. Therefore, determining TED activity and severity accurately is important to guide optimal management.

The clinical activity of TED is frequently assessed by the Clinical activity score (CAS), a composite score of binary measures (yes or no) for different clinical symptoms and signs of inflammation in the anterior orbit (e.g., pain, conjunctival redness, swelling), with a minimum score of 0 and maximum score of 10 [2, 3]. TED severity is also assessed using the European severity scale (ESS) developed by the European Group on Graves Orbitopathy (EUGOGO) which classifies patients into mild, moderate/severe, and sight-threatening groups based on soft tissue signs, eyelid measurement, ocular motility, corneal integrity, and the degree of proptosis [2]. Despite being useful clinical tools, both assessments are observer-dependent and are limited in their assessment of the posterior orbit [4]. The criteria of the scores are binary, which may be too simplistic and not reflect the spectrum of changes that can occur [5].

Magnetic resonance imaging (MRI) is superior to computerised tomography (CT) when it is available for the evaluation of TED due to its superior soft tissue contrast resolution [6, 7]. Besides providing anatomic information concerning muscle and fat measurements and apical crowding, MRI can provide information analogous to active disease by demonstrating changes in signal intensity of involved tissues including EOM [5].

In the evaluation of active TED, the STIR intensity ratio (SIR) plays a crucial role in MRI [8]. STIR sequences are particularly sensitive to fluid content and inflammation, making them well-suited for detecting active disease manifestations such as orbital oedema and inflammation. The SIR, calculated by comparing signal intensities in affected and unaffected orbits, provides a quantitative measure of the inflammatory activity in the thyroid orbit.

Whilst STIR is a valuable tool, diffusion-weighted imaging (DWI) MRI has also gained attention in the assessment of TED. Preliminary research has found significant changes in MRI DWI in patients with TED compared to healthy controls [9]. It has been shown to provide measures of the diffusion of water in soft tissues and non-echoplanar diffusion-weighted imaging (non-EPI DWI) is more resistant to susceptibility artefacts including at bone-air interfaces than echoplanar DWI, which makes it useful for orbital evaluation. Higher apparent diffusion coefficient (ADC) indicates greater facilitated diffusion which can have several mechanisms including tissue oedema or inflammation in benign tissues [9, 10]. We have previously shown the utility of non-echoplanar DWI (non-EPI-DWI) in guiding clinical decisions, especially when the CAS and ESS were unreliable [11].

Previous studies have provided insights into the use of clinical scores and DWI scans in the management of TED [11]; however, these are limited by their sample size. With a larger cohort of patients from a diverse multiethnic population, this study is likely to reflect the situation in clinical practice in terms of encompassing varied presentations of TED and challenges in clinical assessment. This study specifically examines the associations between EOM-ADCs with CAS and ESS as well identify any potential discriminatory cut-offs for clinically useful groups.

## Materials and methods

Patients were identified from three multidisciplinary (MDT) TED clinics at Imperial College Healthcare NHS Trust Hospitals (Western Eye (WEH) and Charing Cross Hospitals (CXH)) and at London North-West University Hospital Trust (Central Middlesex Hospital (CMH)). Anonymised patient databases were evaluated from the inception of the clinics (CMH (Nov 2011), WEH (Jan 2016) and CXH (Jan 2017)) until March 3rd 2021. Patients were selected for analysis based on the inclusion and exclusion criteria as outlined below. The study was approved by the local Audit Committee and adhered to the tenets of the Declaration of Helsinki.

### Inclusion criteria

1. Adult (> 18 years of age).
2. A diagnosis of TED using standard criteria.
3. At least one orbital MRI examination including a non-EPI DWI with corresponding ADC map.
4. CAS within six weeks of MRI scan (or two measurements before and after the scan within three months of the scan).

### Imaging evaluation

MR imaging was performed on a 1.5-T MRI scanner (Magnetom Avanto; Siemens) using a standard Head Matrix coil. In all patients, a 3-mm-thick Half-Fourier Acquisition Single-Shot Turbo Spin-Echo (HASTE) non-EPI DWI sequence was acquired in the coronal plane (Repetition Time (TR) = 900 ms; Echo Time (TE) = 118 ms; matrix = 192 × 86; Field of View (FOV) = 145 mm; 18 averages; EPI spacing = 6.28 ms; bandwidth = 465 Hz/pixel; b factors, 0 and 1000 s/mm^2^). ADC maps were automatically generated by the MRI scanner software. Conventional axial T1 weighted and coronal short tau inversion recovery (STIR) sequences had also been performed and were used for anatomic reference.

### ADC evaluation

**Fig. 1:**
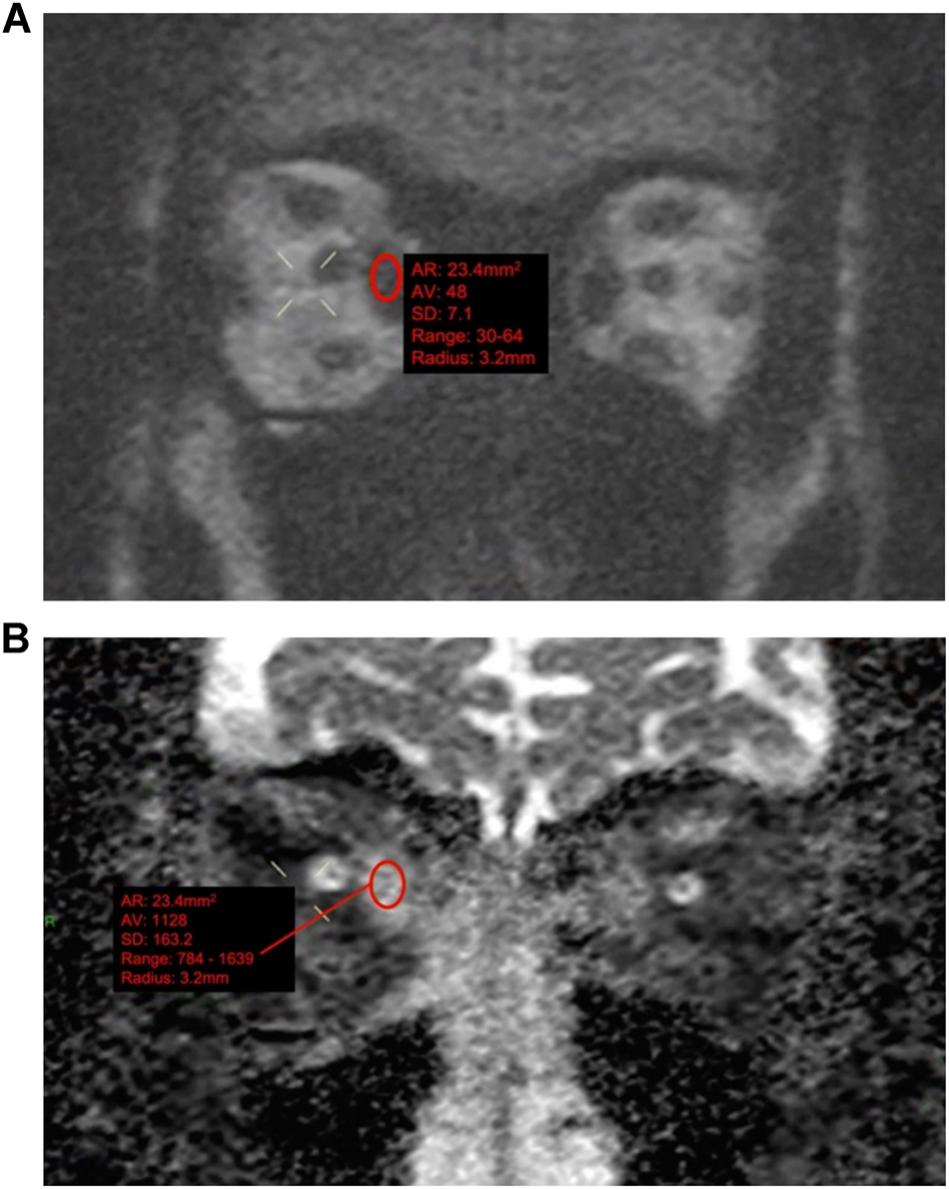
Representative examples of non-echoplanar diffusion-weighted imaging (non-EPI-DWI) of orbital EOMs in a patient with TED. Coronal orbital/EOM non-EPI-DWI MRI scan at a *b* value of 1000 (**A**) and apparent diffusion coefficient (ADC) image (**B**) show moderate-to-marked enlargement of the extraocular muscles and increased ADC values, notably at the right medial rectus (ADC value = 1128). Contour representing the freehand region-of-interest outline within the inner border of the active EOM

A specialist head and neck radiologist (K.B., R.L., G.M.) examined the MR images to select the coronal non-EPI-DWI section that best illustrated EOMs with the highest ADC on visual analysis. This was typically identified in the slice showing the largest diameter of the muscle belly and/or adjacent sections. Using the B0 image initially for optimal muscle delineation (anatomic reference), circular or oval freehand regions of interest (ROIs) were contoured within the inner border of the active EOMs, copied to the ADC map, and the ADC for the ROI was extracted (Fig. 1). The highest EOM-ADC from all the ADCs acquired for the patient was used for subsequent analysis.

### Subgroup analyses

The following subgroups were selected for group-wise analyses since they reflect the clinical classification of patients applied in routine clinical practice.

1. CAS < 3 and CAS ≥ 3 (since CAS ≥ 3 suggest active moderate-to-severe TED requiring systemic immunosuppression [2]).
2. EUGUGO Severity Scale (ESS) [2]: mild, moderate to severe and sight-threatening.

### Receiver operator characteristics (ROC)

To evaluate the potential use of ADC as a diagnostic test for dystrophic optic neuropathy or to exclude mild/possible TED, ROC curves for comparing clinically active vs. inactive TED (i.e., CAS < 3 and CAS ≥ 3).

### Statistical analysis

Statistical analyses were performed using GraphPad Prism Software, Version 9.1.0. Demographic data were summarised descriptively including using median and interquartile range (IQR) as data was non-normally distributed data. To assess for a correlation between EOM-ADC and CAS, which is an ordinal scale with up to ten levels, the Spearman’s Rank correlation test was used (rs, with 95% confidence intervals (CI) and *p*-values). For testing for associations between EOM-ADC and groups classified by CAS and ESS, the Mann–Whitney *U* test was used. Receiver operator curve (ROC) analyses were performed to assess diagnostic performances of EOM-ADCs for differentiating clinical subgroups as described, and diagnostic performance data were recorded for the cut-off achieving the highest overall accuracy. *p*-values < 0.05 were considered statistically significant.

## Results

### Patient characteristics

There were 352 patients (median 46 (range: 36.0–55.0) years; 278 [79.0%] female; 83 [23.7%] current smokers) who attended the three MDTED clinics over the study period. After applying the inclusion criteria, 256 (72.7%) patients were excluded, most of which were due to the absence of a suitable DWI-MRI scan with a concurrent CAS recorded. Ninety-six (27.3%) patients met the inclusion criteria, who had a median age of 50 (range: 39.8–58.3) years, 78.1% were female and 24% were current smokers. Most patients were either hyperthyroid (46.3%) or euthyroid (41.1%) at presentation. The study cohort was ethnically diverse with 34.3% Caucasian, 30.2% Afro-Caribbean and 11.5% Asian. Demographic data are shown in Table 1.

**Table 1:** Patient demographics for radiology cohort analysis

	Radiology Cohort *n* = 96
Age at first TED clinic (years)	
Median (IQR)	50 (39.8–58.3)
No. (%) Female	75 (78.1)
Ethnicity^a^	
No. (%) Caucasian	33 (34.3)
No. (%) Afro-Caucasian	29 (30.2)
No. (%) Asian	11 (11.5)
Smoking Status^b^	
No. (%) current smokers	23 (24.0)
No. (%) never smoked	13 (13.5)
No. (%) ex-smokers	57 (59.4)
No. (%) positive family history^c^	20 (22.2)
Thyroid status at first clinic^d^	
No. (%) Euthyroid	39 (41.1)
No. (%) Hyperthyroid	44 (46.3)
No. (%) Hypothyroid	9 (9.5)
Antithyroid Medication	
No. (%) Carbimazole	34 (37.4)
No. (%) Thyroxine	13 (14.3)
No. (%) IV Methylprednisolone	57 (59.4)
No. (%) Second-line immunosuppression	21 (22.1)
Baseline Clinical Scores at first presentation	
Median CAS (IQR)	2 (1–3)
EUGOGO Severity Scale	
No. (%) Mild	35 (36.5)
No. (%) Moderate-Severe	51 (53.1)
No. (%) Sight-Threatening	10 (10.4)
TSHR Antibody Status	3.95 (155–8.975)

^a^ Data unrecorded *n* = 23,

^b^ Data unrecorded *n* = 3,

^c^ Data unrecorded *n* = 6,

^d^ Data unrecorded *n* = 5,

^e^ Data unrecorded *n* = 5. Data was recorded from the inception of the clinics until March 2021

### Association and correlation between ADC and CAS (disease activity)

**Fig. 2:**
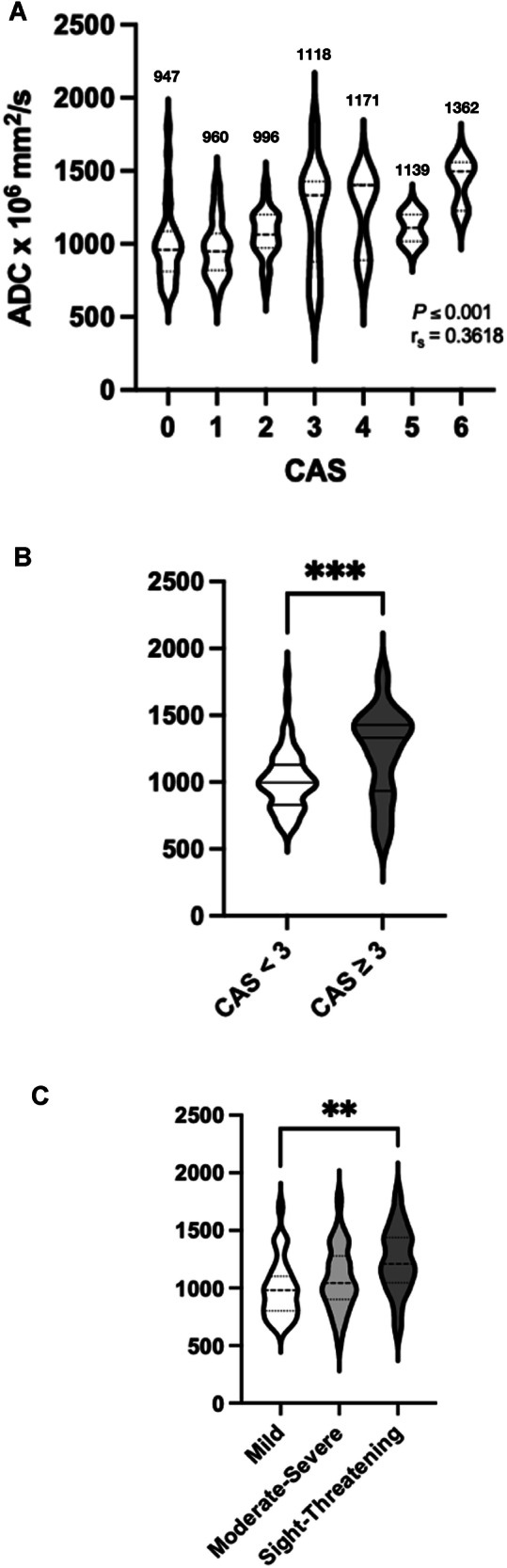
Correlation between CAS and EUGOGO Severity Scale, and ADC with a clinically meaningful group-wise comparison of ADC and CAS in the overall cohort. Violin plots demonstrate a positive Spearman rank correlation coefficient between CAS and ADC (*n* = 105) as well as the median value of each CAS score (in mm^2^/s) (**A**) and significantly greater ADC values in CAS ≥ 3 (*n* = 29) compared with CAS < 3 (*n* = 76) groups (**B**) (***p ≤ 0.001, 2-tailed). Greater ADC values in the sight-threatening severity cohort compared with those in the mild severity cohort (**p ≤ 0.01, 2-tailed) with no significant difference between the other groups (**C**)

The results for EOM-ADCs according to CAS score are shown in Fig. 2 including median values. There was a positive correlation between the EOM-ADC and CAS (r_s_ = 0.362, 95% CI (0.1773–0.5218), *p* ≤ 0.001) (Fig. 2A). Comparing the clinically relevant groups, CAS ≥ 3 (*n* = 29) had a higher ADC (1332 (range: 934–1428) mm^2^/s) than the CAS < 3 (*n* = 76) (997 (range: 830–1131) mm^2^/s) (*p* ≤ 0.001) (Fig. 2B).

### Association between ADC and EUGOGO severity scale (disease severity)

EOM-ADCs along the severity scale of the cohort were as follows: 980 (range: 800–1100) mm^2^/s, 1040 (range: 890–1255) mm^2^/s and 1210 (range: 1044–1438) mm^2^/s for mild (*n* = 35), moderate-severe (*n* = 51) and sight-threatening groups (*n* = 10) respectively. There was a positive trend for a higher ADC according to ESS which was statistically significant between mild and sight-threatening groups (*p* < 0.01) (Fig. 2C).

### ROC analysis

**Fig. 3:**
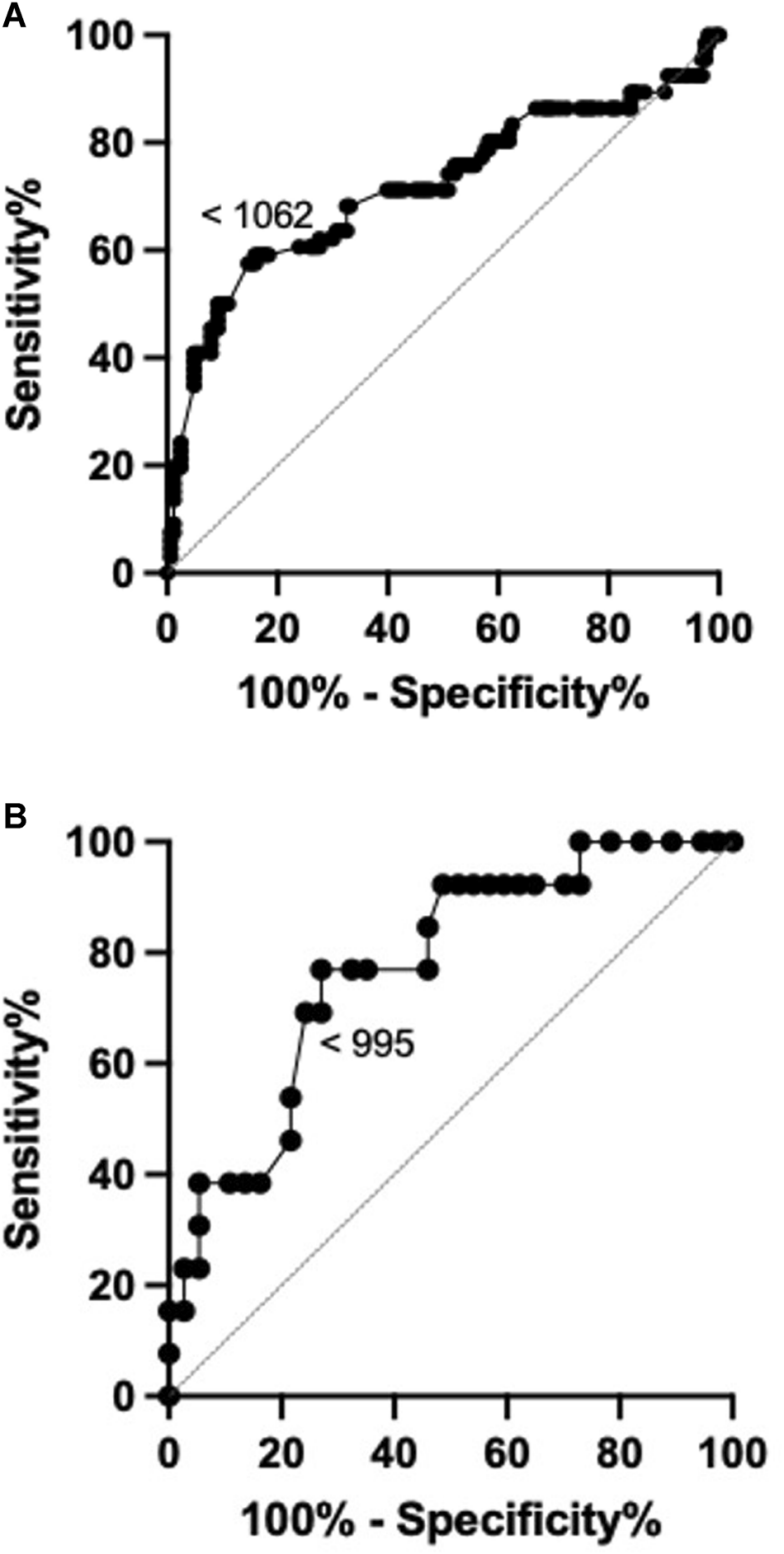
ROCs for the ADC values obtained in subjects with CAS < 3 and CAS ≥ 3 (area under the curve = 0.7178; 95% CI, (0.63–0.80); *p* ≤ 0.0001) (**A**). ROCs for the ADC values were obtained in subjects with mild-moderate disease and sight-threatening disease (area under the curve = 0.7744, 95% CI, 0.63–0.92; *p* ≤ 0.01) (**B**). A diagonal line represents a line of no discrimination between disease states. The arrowed number represents the cut-off ADC values

To differentiate clinically active disease from inactive TED (i.e., CAS < 3 and CAS ≥ 3), an ADC cut-off of 1062 mm^2^/s achieved 60% sensitivity and 72% specificity in diagnosing active disease (area under the curve (AUC) = 0.718, 95% CI 0.63–0.80, *p* ≤ 0.0001) (Fig. 3A). The estimated ADC cut-off to differentiate between mild-moderate disease and sight-threatening disease was 995 mm^2^/s to give 77% sensitivity and 67% specificity (AUC = 0.7744, 95% CI, (0.63–0.92), *p* ≤ 0.01) (Fig. 3B).

## Discussion

CAS and ESS are common clinical scores used to assess the activity and severity of TED [2, 3]. CAS is a useful clinical tool in decision-making for TED patients, however, this clinical measure can over or underestimate true disease activity, even by clinicians with experience [12]. The CAS considers each TED symptom and sign as having equal weighting in scoring and does not include important signs of TED, such as diplopia and proptosis at the initial assessment. The ESS is used to assess the severity of TED which is the functional deficit caused by the disease [2, 3]. This includes the assessment of ocular motility and the clinical proptosis measurement. However, there can be poor intra-observer agreement in clinical proptosis measurements [13]. This raises concerns about the sensitivity of this clinical score as an indicator of TED severity [13]. Furthermore, some patients with functionally severe eye disease may have minimal mild proptosis only, which can be explained in terms of a variation between individuals in how much the orbital septal tissues can be distended axially, and those with less distensible orbital septae may be more likely to develop complications as a result of higher retrobulbar orbital pressures developing similar to a chronic compartment syndrome. Levels of proptosis are also dependent on the ethnicity of the patient which influences the anatomy of the bony orbit [14].

Orbital MRI is an important diagnostic tool in patients with clinically evident TED as it can provide both anatomic and functional imaging information and includes retrobulbar structures (posterior orbit) that are inaccessible to clinical evaluation.

The findings of this study are consistent with previously published data that document a positive association between increased extraocular muscle EOM-ADC values of involved EOM in TED and higher clinical measures of disease activity based on CAS and higher disease severity based on the ESS [6, 7, 10]. Therefore, EOM-ADCs potentially have clinical utility by providing objective quantitative data regarding muscle inflammatory changes in patients with TED, which in turn correlate with disease activity and severity. This is important for several reasons. Firstly, clinical assessment tools currently used to assess disease activity in TED, such as CAS, evaluate signs and symptoms of periorbital inflammation which are binary and inherently subjective. Furthermore, specific features, such as erythema, are difficult to assess in darker-skinned individuals, which in turn limits the reliability of CAS in such cases. In the present study over 40% of patients were non-Caucasian [12, 14, 15]. In addition, CAS does not evaluate the posterior orbit directly, including the EOMs and retroorbital fat, so it can over or underestimate the true disease activity, even if performed by clinicians with experience [12]. For example, some individuals with minimal signs of periorbital inflammation in TED may have severe active inflammation in the EOMs. Consequently, an accurate overview of the activity of TED requires both clinical assessments, e.g. using CAS, and orbital imaging. The present study suggests that EOM-ADCs could be utilised for this purpose.

The underlying pathological mechanism for the elevated ADC measurements in patients with TED is not closely established and could represent increased oedema alone or additional pathophysiological processes [16]. Furthermore, different acute and chronic effects may contribute at different time points. The temporal changes of EOM-ADCs in patients with TED were not addressed in this study, although there is some preliminary data to suggest that ADCs may not normalise in patients in the late phase inactive of TED [11].

In this study, higher ADC values were associated with higher CASs and were higher in the sight-threatening cohort compared to the mild severity group. Although this study showed no statistically significant difference in EOM-ADCs in TED with moderate severity compared to the other groups, it is possible that there are different processes predominating in this group including chronic inflammation and fibrosis, which may have different influences on diffusion in EOMs, which in turn may influence ADCs.

The ROC analyses suggest that EOM-ADC values have the potential to differentiate between clinically active and inactive TED, as well as mild to moderate severity of disease and sight-threatening severity of TED (Fig. 3). The EOM-ADC cut-offs may be used to identify those patients who may require earlier intervention, e.g. immunosuppression therapy for high-risk patients. Furthermore, in patients with apparently low CAS, which reflects minimal anterior orbital symptoms and signs of inflammation, the finding of a very high EOM-ADC, which reflects posterior orbital inflammation, may be used to justify closer clinical monitoring with earlier interval imaging.

### Study limitations

There were several study limitations. First, the DWI-MRI protocol was introduced at different hospital sites at different time points due to the real-life healthcare evolution of the practice. Therefore, many TED patients who had MRI imaging before the protocol introduction had to be excluded from the study. Given the retrospective nature of the study, we had to exclude patients where clinical parameters such as concurrent CAS were not recorded. Second, ADC was calculated by manually drawing ROIs on enlarged EOMs by three experienced radiologists, but at different sites and scanners. Steps were also taken to ensure inter-scan variability was minimised by standardising the imaging parameters, but small differences are likely to remain. This could be improved by scanning the same individual at both sites to evaluate the inter-observer agreement, but this was out of the scope of this study. Previous studies in TED assessment have shown good inter-observer agreement with ADC measurements [8]. Third, there has been some preliminary research into the potential utility of other MRI-derived indices as a surrogate of disease activity including EOM T2 signal intensity, T2 relaxation, and fat fraction estimates [5]. These indices are still experimental and were not evaluated in this study partly due to its retrospective design although it is possible that a combination of MRI indices may have the highest predictive value for disease activity or severity in TED patients.

## Conclusion

Our results show a trend between clinical activity and severity with EOM-ADC across the spectrum of TED. This has clinical utility as EOM-ADC can provide quantitative data pertaining to disease activity in the posterior orbit in TED, which differs from clinically derived measures of disease activity and severity that are subjective and focused on signs of anterior orbital inflammation.

## Data Availability

Patient data was collected from three Thyroid Eye Clinics at different NHS Healthcare Trusts, and was kept anonymous in line with patient confidentiality.

## Abbreviations

ADC: Apparent diffusion coefficient
CAS: Clinical activity score
EOM: Extra ocular muscles
ESS: European Severity Scale
MRI: Magnetic resonance imaging
non-EPI-DWI: Non-echoplanar diffusion-weighted imaging
TED: Thyroid eye disease
